# Chromosomal stability in buccal cells was linked to age but not affected by exercise and nutrients - Vienna Active Ageing Study (VAAS), a randomized controlled trial

**DOI:** 10.1016/j.redox.2019.101362

**Published:** 2019-10-24

**Authors:** Bernhard Franzke, Barbara Schober-Halper, Marlene Hofmann, Stefan Oesen, Anela Tosevska, Armen Nersesyan, Siegfried Knasmüller, Eva-Maria Strasser, Marlies Wallner, Barbara Wessner, Karl-Heinz Wagner

**Affiliations:** a University of Vienna, Research Platform Active Ageing, Althanstraße 14, 1090, Vienna, Austria; b Medical University of Vienna, Institute of Cancer Research, Department of Internal Medicine I, Borschkegasse 8a, 1090, Vienna, Austria; c Karl Landsteiner Institute for Remobilization and Functional Health/ Institute for Physical Medicine and Rehabilitation, Kaiser Franz Joseph Spital, SMZ-Süd, Kundratstraße 3, 1100, Vienna, Austria; d University of Applied Sciences FH JOANNEUM, Eggenberger Allee 11, 8020, Graz, Austria; e University of Vienna, Centre for Sport Science and University Sports, Department of Sport and Exercise Physiology, Auf der Schmelz 6, 1150, Vienna, Austria; f University of Vienna, Faculty of Life Sciences, Department of Nutritional Sciences, Althanstraße 14, 1090, Vienna, Austria; g Department of Molecular, Cell and Developmental Biology, UCLA, 610 Charles E. Young Drive East, Los Angeles, CA, 90095, USA

**Keywords:** Micronuclei, DNA damage, Life-expectancy, Genome stability, Resistance training, Aging biomarker

## Abstract

The purpose of this study was to investigate the effect of six months strength training with or without supplementing protein and vitamins, on chromosomal integrity of buccal cells in institutionalized elderly.

One hundred seventeen women and men (65–98 years) performed either resistance training (RT), RT combined with a nutritional supplement (RTS) or cognitive training (CT) twice per week for six months. Participants’ fitness was measured using the 6 min walking, the chair rise, and the handgrip strength test. Genotoxicity and cytotoxicity parameters were investigated with the Buccal Micronucleus Cytome (BMcyt) assay.

Six minutes walking and chair rise performance improved significantly, however, no changes of the parameters of the BMcyt were detected. Age and micronuclei (MN) frequency correlated significantly, for both women (r = 0.597, p = 0.000) and men (r = 0.508, p = 0.000). Squared regressions revealed a significant increase in the MN frequency of buccal cells with age (R^2^ = 0.466, p = 0.000).

Interestingly and contrary to what was shown in blood lymphocytes, chromosomal damage in buccal cells increases until very old age, which might qualify them as a valid biomarker for aging. Unexpectedly, in this group of institutionalized elderly, resistance training using elastic bands had no effect on chromosomal damage in buccal cells.

## Highlights

• Mutation in buccal cells increased until very old age – a new aging biomarker?
• Chromosomal damage in buccal cells was age-dependent and equal for women and men.
• Strength training improved fitness but not mutagenicity in buccal cells of elderly.

## Graphical abstract

**Figure:**
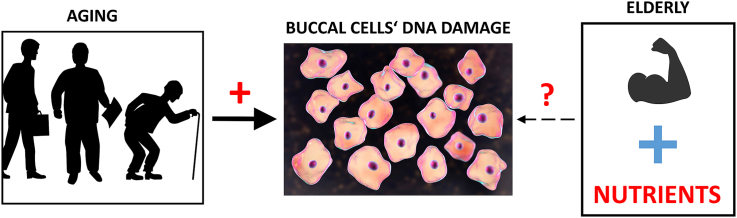

## 1 Introduction

The prevention of age related physical and mental impairment, as well as the reduction of diseases such as Alzheimer's, sarcopenia, diabetes or cancer is one of the main objectives to improve quality of life and reduce the costs for healthcare in the elderly. Increased levels of chromosomal and DNA damage are associated with the development of these chronic diseases [1] and further accompanied by the loss of strength, physical function and muscle mass [2,3] in the elderly. The change of chromosomes, caused by aggressive reactive oxygen species and other factors, can lead to cellular death (apoptosis or necrosis), further to tissue and organ malfunction and finally to diseases and/or accelerated mortality [4,5]. Successful strategies to increase chromosomal stability and improve the resistance of cells against oxidative stress are based on lifestyle modifications including physical activity and the diet [6,7]. Thus, the combination of resistance training and protein seems to be most effective in increasing physical fitness and muscle mass in the elderly [8].

The frequency of micronuclei (MN) in blood lymphocytes is a well-established marker to measure chromosomal damage [9]. Interestingly, it has been shown, that very old, successfully aged humans, beyond their statistical life-expectancy, have lower MN frequencies than younger elderly (60–70 years of age), which are comparable to young adults [10,11]. At present, there are only data available about elderly and lifestyle interventions investigating MN frequencies in peripheral blood lymphocytes, but not in buccal cells. Exfoliated buccal cells however, are an interesting material, as their sampling is, contrary to blood lymphocytes, non-invasive [12] and they might be of high relevance in future since up to 90% of all cancers are of epithelial origin [13].

The primary aim of this secondary analysis of the Vienna Active Ageing Study was to investigate the effect of a six months lifestyle intervention, including strength training (using elastic bands) and the intake of a nutritional supplement (proteins and vitamins), on markers of the Buccal Micronucleus Cytome (BMcyt) assay in Austrian institutionalized elderly women and men. We hypothesized improvements in chromosomal stability through exercise with further enhanced effects in subjects, who took the supplement.

A secondary aim was to further investigate the link between age and parameters of the BMcyt by combining our data with those of Wallner et al. [14], who analyzed younger subjects in the same laboratory.

With the age of our study subjects (women 82.86 years; men 84.85 years) being at or above life expectancy in Austria and 75% of our study population being 80 years or older, we were able to generate novel data on chromosomal damage using the BMcyt assay.

## 2 Material and methods

The presented data are secondary analyses and part of the Vienna Active Ageing Study, which is a multidisciplinary project of the Centre for Sport Science and University Sports, the Faculty of Life Sciences, the Research Platform Active Ageing (all University of Vienna), the Karl Landsteiner Institute for Remobilization and Functional Health (Institute for Physical Medicine and Rehabilitation) and the Curatorship of Viennese Retirement Homes. The study was approved by the ethics committee of the City of Vienna (EK-11–151–0811) and registered at ClinicalTrials.gov, NCT01775111.

### 2.1 Subjects

**Fig. 1:**
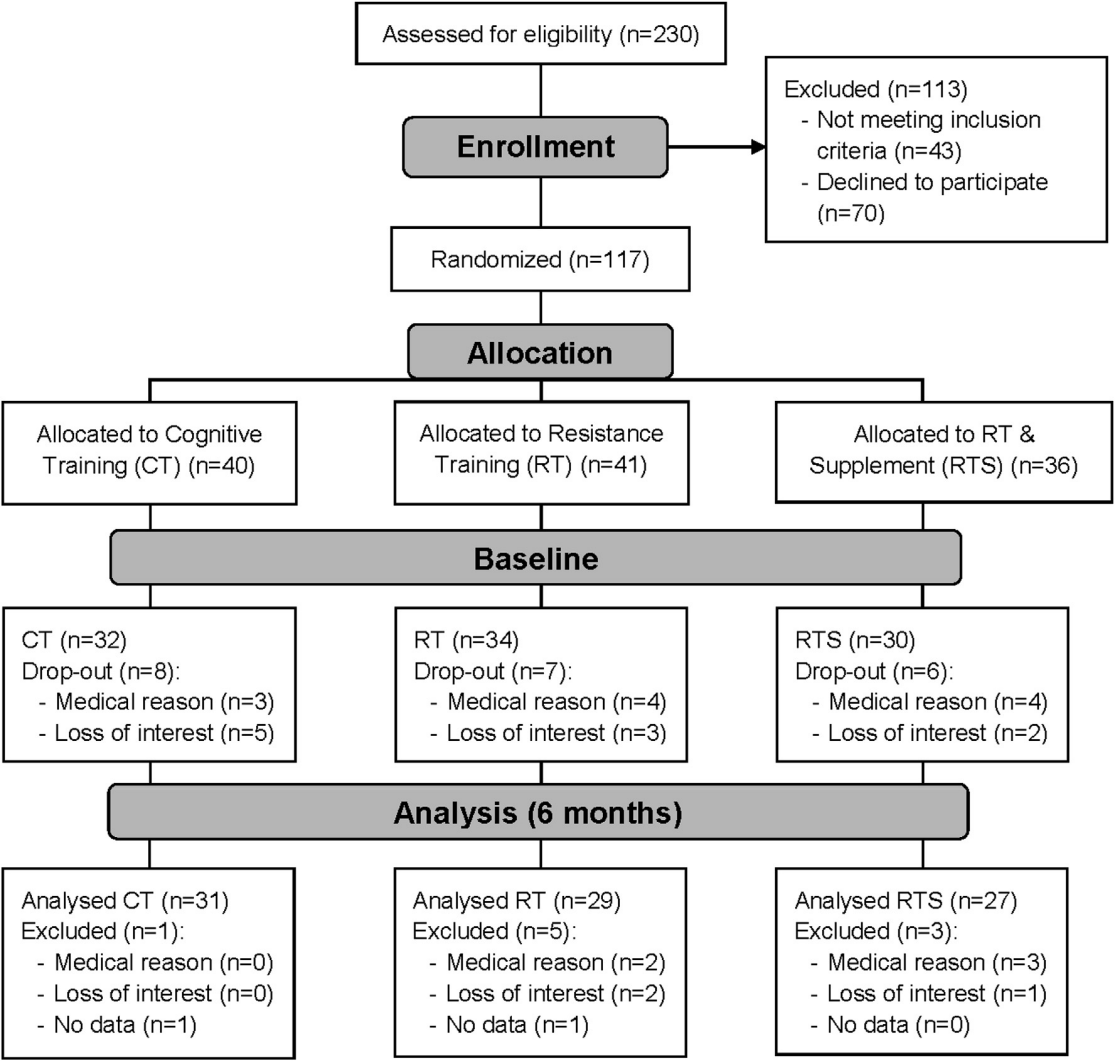
Participants flow diagram.

One hundred seventeen institutionalized elderly women and men (aged 65–98 years), recruited from five different senior residencies in the area of Vienna (Curatorship of Viennese Retirement Homes), volunteered for the study (Fig. 1). The subjects were mentally (Mini Mental State Examination ≥ 23) and physically (Short Physical Performance Battery > 4) able to participate in this training intervention study. They were sedentary (less than 1 h of physical activity or exercise per week) and free of severe diseases that would contra-indicate medical training therapy or measurement of physical performance, including cardiovascular diseases, diabetic retinopathy and regular use of cortisone-containing drugs. Inclusion and exclusion criteria have been described previously [15]. The health condition of all study subjects was assessed by specialists in internal medicine and gerontology. Written informed consent was obtained from all participants before entry into the study in accordance with the Declaration of Helsinki. Subjects were not allowed to take part in any exhausting physical activity within 2 days before the blood sampling and fitness test. All participants followed their medication protocols as prescribed by their physicians. If supplements were consumed before entering the study, details on further intake were discussed with their physicians.

### 2.2 Study design

The present study design was described previously in Franzke et al. [16]. Briefly, study participants were randomly assigned into three intervention groups – cognitive training (CT), resistance training (RT), RT + supplement (RTS) – and matched for gender in a randomized, controlled, observer-blind design. At baseline (T1) and after six months (T2) blood samples were taken as well as physical and functional tests were performed. The effects of either a resistance training intervention, a resistance training and nutritional supplementation intervention or a cognitive training intervention (serving as a control group for the physical training groups) on markers of the BMcyt assay in institutionalized elderly were assessed. We further investigated the influence of age on these markers of chromosomal damage.

### 2.3 Resistance training

The resistance training groups (RT and RTS) received two weekly sessions of resistance training, conducted on two non-consecutive days and supervised by a sport scientist. Training attendance was recorded every session. Exercises were conducted using elastic bands, chairs and own body weight - for detailed training program see supplement of Oesen et al. [15]. The progressive resistance training protocol was designed based on the guidelines of the American College of Sports Medicine for resistance training with older subjects [17]. The about 1 h lasting workout consisted of an initial 10 min warm up, a 30–40 min strength training for the main muscle groups (legs, back, abdomen, chest, shoulder and arms) and a 10 min cool down. To keep the training stimulus effective, the exercise program was adjusted to the participants’ individual needs, by either adapting the resistance of the elastic band (shorter or stronger band) or by modifying the exercise, by means of performing a more difficult version. In the initial phase (4 weeks) one set of 15 repetitions was performed in order to learn the correct form of each exercise. From the fifth week on, training intensity and volume were progressively increased from two sets of light exercises to two sets of heavy resistance. If the participants could easily perform two sets of 15 repetitions they were asked to either take more resistance or to perform a more difficult version of the exercise.

### 2.4 Resistance training and supplementation

The RTS group performed the same exercises together with the RT group and additionally a liquid supplement every morning, as well as directly after each training session. Each drink supplied a total energy of 150 kcal and contained 20.7g protein (56 energy (En)%, 19.7g whey protein, 3.0g leucine, > 10g essential amino acids), 9.3g carbohydrates (25 En%, 0.8 BE), 3.0g fat (18 En%), 1.2g roughage (2 En%), 800IU (20 μg) of vitamin D, 250 mg calcium, 31.5 mg vitamin C, 7.5 mg vitamin E, 0.8 mg vitamin B6, 3 μg vitamin B12, 199.5 μg folic acid and 37.5 mg magnesium (FortiFit, NUTRICIA GmbH, Vienna, Austria). The intake of the nutritional supplement was controlled at breakfast (prepared by the kitchen staff and served ready-to-drink for the participants) as well as after the training sessions, where the trainers mixed the supplement and distributed it to the participants.

### 2.5 Cognitive training

The CT groups served as our control group and performed coordinative or cognitive tasks two times per week, equally to the frequency of the RT and RTS groups. This was done to minimize the “bias” of being part of a social group activity.

Participants of all groups were instructed to maintain their regular food intake.

### 2.6 Buccal Micronucleus Cytome (BMcyt) – assay

All study participants were asked to rinse mouth twice thoroughly with 100 ml of tap water to remove excess debris. Then the participants used toothbrushes and rubbed them on the insides of both cheeks to collect buccal cells, which were processed and scored according to the method of Thomas et al. [12]. 80 000 cells/mL were transferred to slides and a minimum of 2000 differentiated cells/slide were counted to ensure accuracy of the results. For staining with Feulgen, cells were placed in beakers with 5.0 M HCl at room temperature for 15 min, rinsed with distilled water (30 min) and subsequently stained with Schiff's reagent (90 min). Cells were scored under bright field with 400-fold magnification using Eclipse E600 microscopes (Nikon, Japan) and then confirmed as positive under fluorescence. MN were scored combining both basal and differentiated cells, according to the criteria defined by Thomas et al. [12]. The analysis of the slides was carried out by one experienced scorer and cross-checked by another experienced scorer.

### 2.7 Chair rise test

The participants had to stand up from a chair (46 cm seat height) as often as possible within 30 s. The chair was placed backwards against a wall to ensure a safe test setting. For one valid repetition, participants had to fully stand up (hip and knee fully extended) and sit back, with their arms crossed over their chest. A last-second-attempt was considered valid, if the person had covered more than 50% of the range of motion [18].

### 2.8 Handgrip strength test

All participants performed an isometric handgrip strength test (kg) using a dynamometer. The test was conducted in a sitting position and maximal isometric contraction within 4–5 s was measured (JAMAR compatible handgrip dynamometer adapted to handle different sizes, Lafayette Instrument, USA, Lafayette). The better result of two trials (1 min break in between) for each hand was recorded [19].

### 2.9 Six minutes walking test

The participants had to walk for 6 min as fast and as far as possible. This test is a valid tool to evaluate aerobic endurance in the elderly. Participants were allowed to slow down and even take short rests. Every subject performed the test separately without being disturbed by others. They had to walk back and forth on a 30 m shuttle track and the distance covered within 6 min was registered [20].

### 2.10 Statistics

Statistical analyses were performed with IBM SPSS Statistics 21. After testing for distribution of the data, baseline differences were measured using the Mann-Whitney-U test. Linear and curve fitting regressions were calculated for MN-frequency and age. To analyze age effects, additional data generated by our working group were included [14]. Both, the present study and the study of Wallner et al. [14], performed the BMcyt-assay according to the protocol of Thomas et al. [12]. Friedman- and, if significant differences were found, the Wilcoxon-Test were performed to calculate the differences between the time points. A p-value ≤ 0.05 was considered significant. An a priori power analysis showed that at an α of 0.05/3 and a power of 0.85 a total number of 86 participants would be necessary to detect changes in functional and strength parameters (isometric strength - leg extension and flexion, 6 min walking test, chair rise test). With an estimated drop-out number of 40% over 6 months we aimed at starting the study with 120 subjects.

### 2.11 Data availability

Underlying data for this publication can be accessed via the following source - https://figshare.com/s/c4c095d491ad1c33f15e.

## 3 Results

### 3.1 Demographic characteristics

At baseline, 96 subjects were included into the Active Aging study (Figs. 1), 12.4% were males, 87.6% females, which constitutes a representative gender distribution for the houses of the Curatorship of Viennese Retirement Homes. The participants had a mean age of 83.0 ± 6.1 years and a BMI of 29.3 ± 5.0 kg/m^2^. Baseline analyses for the BMcyt assay were performed for the 87 subjects who participated in the six months sampling time-point.

There was no statistical difference between women and men for the parameters of the BMcyt, age and BMI, however the number of cells with MN (p = 0.060) and the total number of MN (p = 0.064) showed a tendency for increased frequencies in female subjects (women: MN cells 1.23 ± 0.44, MN total 1.34 ± 0.47; men: MN cells 1.00 ± 0.22, MN total 1.09 ± 0.30).

Data regarding the physical performance of our study population have been published in detail by Oesen et al. [15]. These results demonstrate the relatively poor functional and physical fitness of the participants with 104 ± 37% of the reference performance in the chair rise test for their age-group, 90 ± 30% in the handgrip strength test and only 68 ± 18% in the 6 min walking test compared to normative values for this age group [[21], [22], [23]].

### 3.2 MN and age

To assess the effect of age on the parameters of the BMcyt assay, we statistically merged our data with the study of Wallner et al. [14] who investigated younger subjects in the same laboratory. A combined sample size of 163 subjects between 20 and 98 years of age was achieved. Age correlated significantly with all parameters of the BMcyt assay, except for pyknotic cells (MN cells: r = 0.682, p = 0.000; MN total: r = 0.647, p = 0.000; nuclear buds: r = 0.310, p = 0.000; binucleated cells: r = 0.631, p = 0.000; karyorrhectic cells: r = 0.593, p = 0.000; karyolytic cells: r = 0.505, p = 0.000; pyknotic cells: r = 0.032, p = 0.686).

**Fig. 2:**
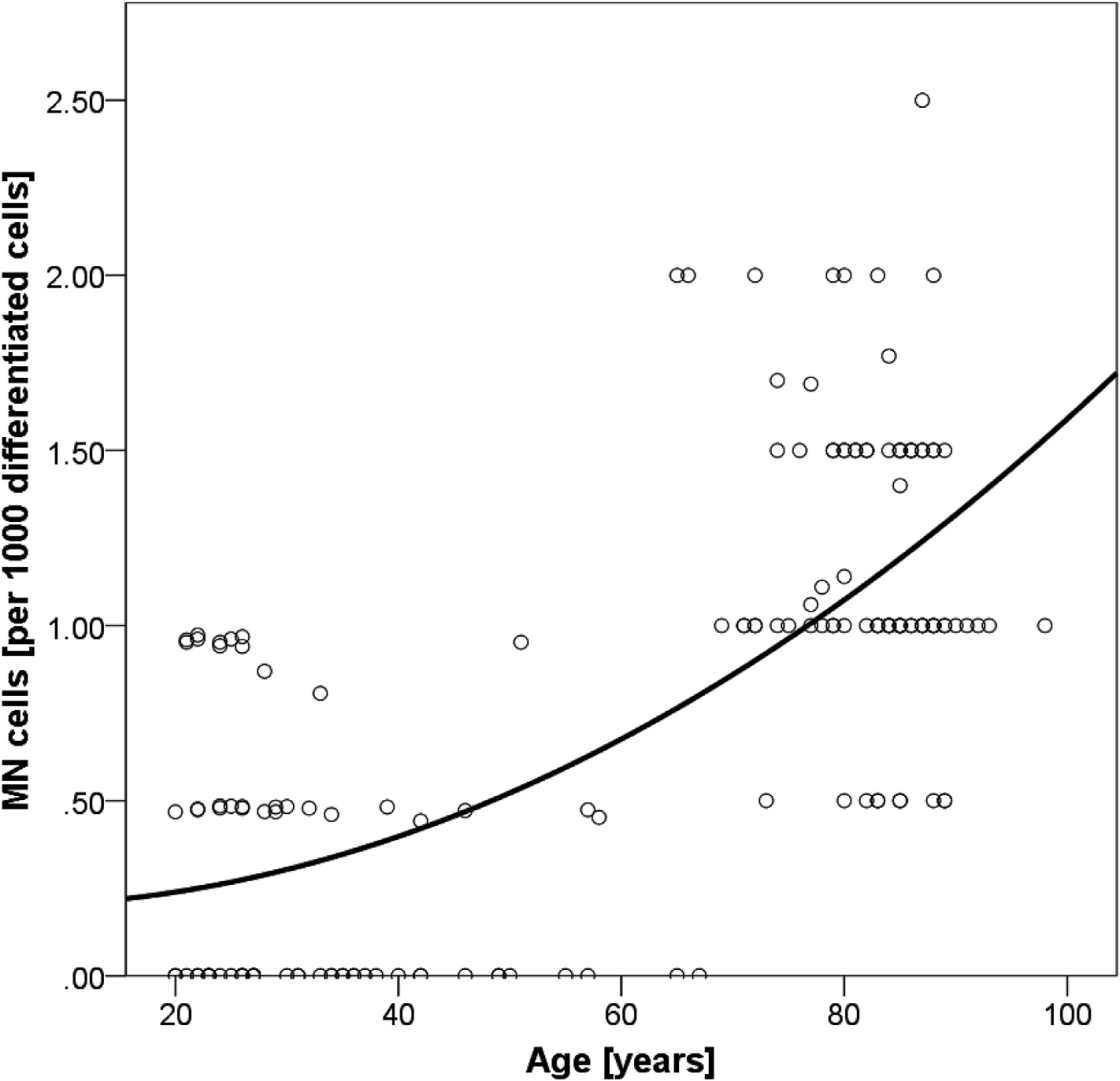
Squared regression between age and buccal MN frequencies (R^2^ = 0.466, p = 0.000).

**Fig. 3:**
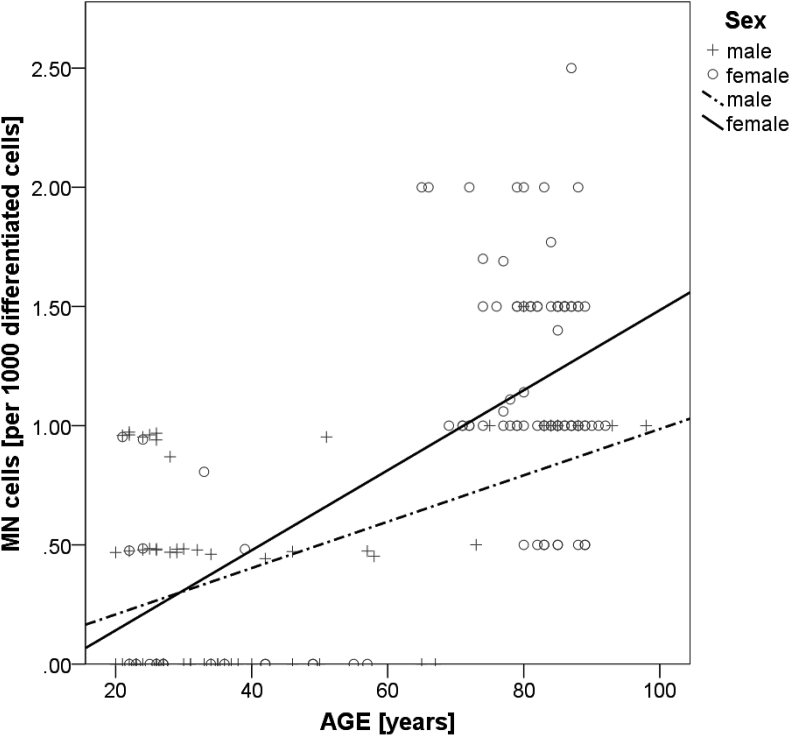
Linear correlations between age and MN frequency for women and men. (women: r = 0.597, p = 0.000; men: r = 0.508, p = 0.000).

A detailed analysis of the MN frequency showed a significant squared regression between MN cells and age (R^2^ = 0.466, p = 0.000) (Fig. 2). For both, women and men a significant correlation between age and MN cells was demonstrated, with no statistical gender difference (women: r = 0.597, p = 0.000; men: r = 0.508, p = 0.000) (Fig. 3).

Baseline data of age, BMI and the BMcyt assay are presented in Table 1. Older subjects (60 years and older; mean age = 82.8 ± 6.5 years) showed significantly higher values in all parameters compared to the younger ones (under 60 years; mean age = 31.2 ± 10.1 years), except for pyknotic cells.

**Table 1:** Baseline demographic characteristics of age, BMI and nuclear anomalies in buccal cells.

Parameter	All	Young (20–59.9 years)	Old (60 + years)	P
Subjects [number]	163	74	89	**0.000**
Age [years]	61.7 (±26.7)	31.2 (±10.1)	82.8 (±6.5)	**0.000**
BMI [kg/m^2^]	26.84 (±5.14)	23.6 (±3,2)	29.2 (±5.0)	**0.000**
*Buccal Micronucleus Cytome Assay*				
MN cells [per 1000 diff. cells]	0.76 (±0.61)	0.27 (±0.36)	1.17 (±0.46)	**0.000**
MN total [per 1000 diff. cells]	0.85 (±0.66)	0.34 (±0.44)	1.28 (±0.49)	**0.000**
Nuclear buds [per 1000 diff. cells]	0.88 (±0.78)	0.58 (±0.56)	1.13 (±0.84)	**0.000**
Binucleated cells [per 1000 diff. cells]	18.7 (±9.3)	12.0 (±4.8)	24.2 (±8.6)	**0.000**
Karyorrhectic cells [per 1000 cells]	16.9 (±10.5)	9.6 (±5.6)	22.9 (±9.9)	**0.000**
Karyolytic cells [per 1000 cells]	46.5 (±29.0)	29.7 (±23.8)	59.9 (±25.8)	**0.000**
Pyknotic cells [per 1000 cells]	1.24 (±0.96)	1.23 (±0.92)	1.25 (±1.00)	0.195

Data are means ± SD; p-values for differences between “Young” and “Old” subjects were calculated using Mann-Whitney-U-Test; significant differences are highlighted using bold formatting. For this table data from the Vienna Active Ageing Study and Wallner et al. ^(14)^ were combined.

### 3.3 BMcyt assay parameters and physical fitness

To assess the effect of physical fitness on chromosomal damage and cytotoxicity, we compared the functional fitness parameters (6 min walking test, the chair rise test and the handgrip strength test) with the endpoints of the BMcyt assay. We found no association between chromosomal stability, cytotoxicity parameters and physical fitness. Still, karyolytic cells correlated negatively with the chair raise test (r = −0.267, p = 0.017) and the 6 min walking test (r = −0.271, p = 0.013).

### 3.4 Effects of the intervention on BMcyt parameters

After six months of intervening with RT alone, with RT combined with a nutritional supplement or with cognitive training, we did neither observe statistically significant differences of the BMcyt parameters to baseline, nor between intervention groups (Table 2).

**Table 2:** Impact of 6 (T2) months of intervention on nuclear anomalies in buccal cells.

Intervention	RT			RTS			CT		
	T1	T2	P	T1	T2	P	T1	T2	P
Subjects [number]	29	29		27	27		31	31	
MN cells [per 1000 diff. cells]	1.25 ± 0.45	1.31 ± 0.62	0.673	1.24 ± 0.47	1.22 ± 0.51	0.840	1.12 ± 0.35	1.13 ± 0.65	0.878
MN total [per 1000 diff. cells]	1.35 ± 0.46	1.55 ± 0.63	0.071	1.41 ± 0.50	1.09 ± 1.85	0.119	1.18 ± 0.39	1.31 ± 0.69	0.250
Nuclear buds [per 1000 diff. cells]	1.22 ± 0.81	1.31 ± 0.75	0.797	1.20 ± 0.97	1.16 ± 0.68	0.563	1.03 ± 0.75	1.03 ± 0.76	0.982
Binucleated cells [per 1000 diff. cells]	26.1 ± 9.6	28.7 ± 9.5	0.066	24.9 ± 7.4	25.5 ± 9.3	0.828	22.7 ± 8.0	25.3 ± 7.8	0.052
Karyorrhectic cells [per 1000 cells]	22.9 ± 9.8	25.3 ± 9.1	0.105	24.1 ± 8.7	26.3 ± 7.5	0.071	22.5 ± 10.7	25.1 ± 11.0	0.072
Karyolytic cells [per 1000 cells]	59.8 ± 26.8	62.9 ± 27.2	0.385	55.4 ± 22.8	54.1 ± 21.1	0.691	66.5 ± 25.3	67.1 ± 24.6	0.717
Pyknotic cells [per 1000 cells]	1.38 ± 1.15	1.55 ± 1.06	0.373	0.89 ± 0.80	1.07 ± 0.73	0.330	1.45 ± 0.99	1.35 ± 0.84	0.528

Data are means ± SD; p-values were calculated using Wilcoxon-Test. Only data from the Vienna Active Ageing Study were used for the analyses.

Both, RT and RTS groups significantly improved in the chair rise test and the 6 min walking test, but not in the handgrip test (RT: chair rise: +17%, p = 0.006; 6 min walking: +6%, p = 0.004; RTS: chair rise: +19%, p = 0.002; 6-min-walking: +8%, p = 0.029); for further details please see Franzke et al. [16] and Oesen et al. [15].

## 4 Discussion

The primary aim of the current secondary analysis of the Vienna Active Ageing Study was to investigate the effect of six months strength training, strength training combined with a protein-vitamin supplement, or of cognitive training on parameters of chromosomal instability and/or various stages of cell death, measured by the BMcyt assay, in a cohort of elderly institutionalized women and men in Vienna. Previous research on MN in human PBMCs [24] guided our attention towards the effect of age on genome stability. To further expand the age-span of our analyses, we merged our data (subjects between 65 and 98 years) with data of Wallner et al. [14], who analyzed MN in buccal cells (analyses performed in the same laboratory) in a younger group.

Our study cohort of institutionalized elderly (women 82.9 ± 6.0 years; men 84.9 ± 6.7 years) with a mean age around their current statistical life-expectancy [25] reflects the current situation in retirement homes in Vienna, with a gender distribution of 85% women and only 15% men [26,27]. BMI values were higher in older subjects (Table 1) and with an average of 29 kg/m^2^ classified as “overweight”, which is rather supportive than detrimental for this advanced age group. In elderly a higher bodyweight was observed to be inversely associated with mortality [28] and even linked to a reduced sarcopenia risk [29].

The MN frequency in buccal mucosa cells correlated positively with age in healthy individuals in earlier studies and equally for both sexes [30,31], which is in agreement with our findings (Fig. 3).

Our merged data, with results from 163 subjects between 20 and 98 years, showed a significant squared regression between age and MN frequencies (Fig. 2), with more chromosomal aberrations and increased parameters of cytotoxicity the higher the age of our subjects was. Interestingly, in lymphocytes this increase was not observed in the same subjects; for details please see Franzke et al. [24]. It was postulated that chromosomal damage in lymphocytes reaches a maximum at the age of 60–70 years [11,32], and then plateaus or even decreases with further aging, leading to a cohort of superior resilient, very old “super-agers” who show cellular characteristics normally found in younger cohorts [7,33]. However, buccal cell metabolism seems to be more stable and not to be affected by these cellular and genetic mechanisms as their MN frequency increases with age without leveling-off [30,31,34]. Contrary to what was shown in lymphocytes and buccal cells, aging seems not to affect MN frequency in human fibroblasts, although DNA damage was positively associated with age [35]. There is definitely need for further research activities on MN frequency dynamics in different tissues in the context of aging, yet buccal cells seem to be highly promising: 1) collecting buccal cells is the least invasive method, compared to PBMCs and fibroblasts; 2) genome stability in buccal cells steadily increases until very old age, which is not always seen in other tissues/cell types.

To our knowledge there are no published studies investigating the effect of exercise on MN formation in buccal cells. Exercise with its high oxygen demand increases the formation of reactive oxygen species, which, under normal physiological conditions, serve as essential signaling molecules in the adaptation process to physical training [36]. However, frequently performed long, intense and unaccustomed exercise training could induce chronically elevated oxidative stress, causing deterioration in DNA stability and finally leading to damaged DNA [36], cellular senescence and chronic inflammation [37]. By contrast, regular exercise was found to have a potential DNA-protective effect by up-regulating antioxidant enzyme activity and DNA repair mechanisms [38,39], which have been shown to reduce MN frequency, yet only in lymphocytes [40].

We did not observe any correlations between MN frequency in buccal cells and physical performance parameters in our study cohort at baseline. After six months of strength training alone or in combination with the intake of a supplement containing protein and vitamins no effect on MN frequency and other parameters of the BMcyt assay was observed.

Several explanations may account for the lack of an effect of the exercise intervention. Our subjects were, relatively healthy, free of severe diseases and without severe nutrient deficiencies. In buccal mucosa cells it has been shown, that folate is linked to reduced MN frequencies, however this was only the case in folate deficient or diseased populations [41,42]. Although we observed a link between improved chromosomal stability in lymphocytes and increased plasma vitamin B12 in the RTS groups, there was no link to increased folate [24]. The significantly shorter lifespan of buccal cells – max. 28 days [12] – could have blurred the potentially positive long time effect of the intervention, as acute changes in the participants’ health status, which are common in elderly aged around their life-expectancy, can have much stronger effects on this tissue, compared to lymphocytes with a half-life in the human body of more than two years [43,44].

It is possible that our cohort of institutionalized elderly was probably and despite their high age “too healthy” to detect an effect of our interventions. Further, the high age of our subjects (around their statistical life-expectancy) produced a naturally given relatively heterogeneous study group, although we followed strict in- and exclusion criteria. Consequently, in future projects, an even larger sample size (if possible) would be helpful to investigate effects of lifestyle interventions on parameters of the BMcyt assay.

## 5 Conclusion

Despite observing no effect of the exercise and nutritional intervention regarding chromosomal stability in buccal cells of our oldest subjects, our baseline analyses of the combined cohorts (old and young) revealed interesting results. We observed a consistent increase in parameters of the BMcyt assay until very old age (90 years plus), yet contrary to what was seen in lymphocytes, where a leveling off after the age of 60–70 years in the MN frequency occurred. This steady increase in the MN frequency until old age was equally strong for women and men and could, after further research, qualify this marker as a valid biomarker for aging [45].

Our analyses of chromosomal damage in buccal cells, in a cohort of institutionalized elderly around their statistical life-expectancy, add important and missing data to the field of DNA stability and mutagenesis. To our knowledge, we are the first to report about the effect of a lifestyle intervention in elderly, including nutrition and exercise, on parameters of the BMcyt assay.

However, in general and especially in this steadily growing population of elderly people, more research is needed to investigate the underlying mechanisms and tissue related differences regarding chromosomal and DNA integrity to revise and develop further strategies for improved quality of life.

## Funding

This work was supported by the University of Vienna, by funding the Research Platform Active Ageing, the Anniversary Fund of the Austrian National Bank (No. 14541) and the EU-IRSES-318962—BIOAGE project. This article was supported by the Open Access Publishing Fund of the University of Vienna.

## Declaration of competing interest

The authors declare that there is no conflict of interest.
